# Lidocaine represses proliferation and cisplatin resistance in cutaneous squamous cell carcinoma *via* miR-30c/SIRT1 regulation

**DOI:** 10.1080/21655979.2022.2031419

**Published:** 2022-02-25

**Authors:** Tao Liu, Fei Jiang, Li-Yuan Yu, You-Yang Wu

**Affiliations:** Department of Anesthesiology, Xiangyang Central Hospital, Affiliated Hospital of Hubei University of Arts and Science, Xiangyang, Hubei, China

**Keywords:** Lidocaine, cisplatin resistance, cutaneous squamous cell carcinoma, MiR-30c, cancer therapy, drug resistance

## Abstract

This study aimed to determine the effects of lidocaine on cell proliferation and cisplatin resistance in A431 human cutaneous squamous cell carcinoma (cSCC) cells and elucidate the underlying mechanism. Cell proliferation, colony numbers, and cisplatin resistance were determined in A431 or cisplatin-resistant A431 (A431-R) cells that were first transfected with miR-30c-inhibitor or miR-30c-mimic, respectively, and then treated with different concentrations of lidocaine, cisplatin, or both. The expression levels of miR-30c and Sirtuin 1 (SIRT1) in A431 and A431-R cells were determined by quantitative real-time polymerase chain reaction and Western blotting. Lidocaine suppressed A431 cell proliferation and cisplatin resistance in a dose- and time-dependent manner *via* the miR-30c/SIRT1 pathway. MiR-30c overexpression also suppressed cell proliferation and cisplatin resistance in A431 cells by directly targeting and downregulating SIRT1, thus enhancing the protective effects of lidocaine. Conversely, SIRT1 upregulation or miR-30c inhibition antagonized the inhibitory effects of lidocaine. Our results suggest that lidocaine may suppress the progression of cSCC by activating the miR-30c/SIRT1 pathway, indicating its promising potential as a treatment strategy for cSCC.

## Graphical Abstract

**Figure:**
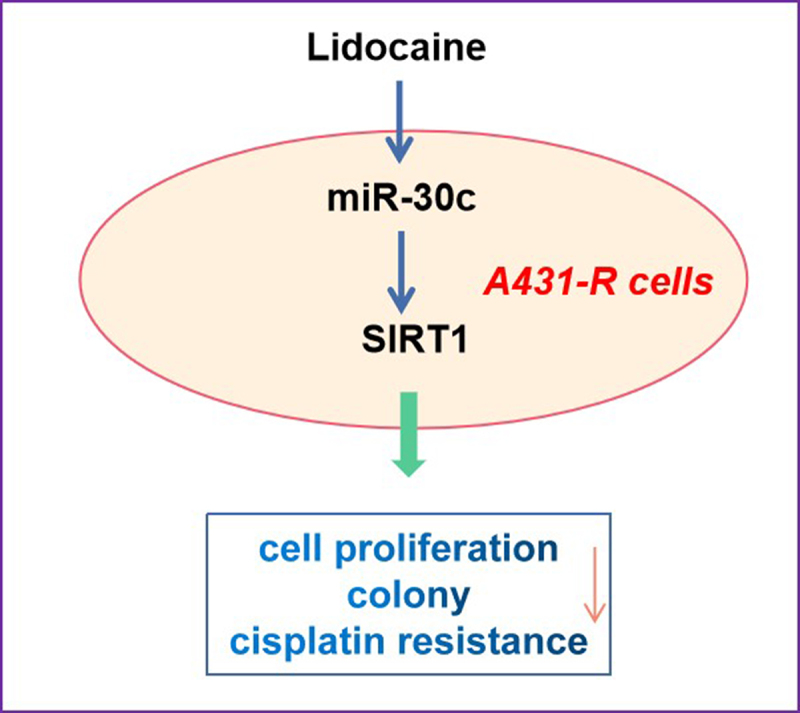

## Introduction

Cutaneous squamous cell carcinoma (cSCC) is an epidermal keratinocyte-derived non-melanoma skin tumor that is affected by multiple factors, including chronic sun exposure, older age, fair skin, immunosuppression, and actinic keratosis [1]. Although cSCC shows benign clinical features due to local invasion and metastasis [2], it remains the second most common tumor in humans worldwide, with already high incidence and mortality rates that are expected to double by 2030 [3]. Previous study reported that several molecular pathways have been implicated in cSCC development, such as CDKN2A, NOTCH, RAS, MAPK, and PI3K/AKT/mTOR pathways [4]. However, there is still a lack of targeted drugs for cSCC treatment. Therefore, there is an urgent need to elucidate the pathogenesis of cSCC and develop effective therapeutic strategies against it.

Several pathways and molecules, such as microRNAs (miRNAs), have been associated with cSCC progression. MiRNAs are endogenous, small, non-coding RNAs of 19–25 nucleotides that modulate gene expression at the post-transcriptional level by binding to the 3’-untranslated region (3’-UTR) of target mRNAs, thereby inhibiting their translation or triggering their degradation. Dysregulated miRNA expression has been associated with the occurrence and progression of many tumors, including cSCC [5]. Exosomal miRNA released by cancer cells can mediate phenotypical changes in the cells of tumor microenvironment to induce tumor growth and therapy resistance [6]. MiRNAs have been found to target important oncogenes such as MYC, RAS family members (HRAS, KRAS and NRAS) and high-mobility group AT-hook2 (HMGA2) to suppress tumor growth [7]. Mechanistically, miRNAs involves in cell proliferation, differentiation, survival, metabolism, genome stability, inflammation, invasion and angiogenesis to affect tumor development [7].

MiR-30c, which is expressed in various malignant cancer tissues, can regulate immunosuppression and resistance to programmed cell death protein 1 in hepatocellular carcinoma, while it has been found to suppress prostate tumor growth by targeting the oncoprotein alternative splicing factor/splicing factor 2 pathway [8]. Endothelial miR-30c has been reported to suppresse tumor growth through inhibiting TGF-beta-induced Serpine1 [9]. Additionally, miR-30c inhibits prostate cancer survival by targeting the ASF/SF2 splicing factor oncoprotein [10]. Moreover, underexpression of miR-30c may promote cSCC development [11]. Clarifying the role of miR-30c in cSCC may help identify novel treatments.

Sirtuin 1 (SIRT1) is a protein deacetylase that regulates the activity of several proteins implicated in cancer development and progression [12]. SIRT1 overexpression has already been found in several tumor cells, including acute myeloid leukemia (AML) and primary colon, prostate, melanoma, and non-melanoma skin cancers, whereas SIRT1 low-expression was demonstrated in breast cancer and hepatic cell carcinomas [13]. In tumor, some researchers have shown the opposite effects of SIRT1 as an oncoprotein or a tumor suppressor under different conditions. In the tumor microenvironment, both infiltrated immune cells and cancer cells can be affected by SIRT1 [13]. Interesting, some miRNAs can regulate the expression of SIRT1 to change the cancer cells growth. For example, miR-199a-5p has been found to directly target SIRT1, thus repressing the stemness of cSCC stem cells [14]. Whether miR-30c regulates cSCC cell survival by targeting SIRT1 is unclear.

Recently, lidocaine is widely used as a local anesthetics, and also exerts multiple other roles. Extensive observations reported that lidocaine can ameliorate the neurological outcome and repress the incidence of spinal cord injury in vivo [15,16]. Additionally, many investigations have demonstrated that lidocaine might show anti-tumor effects on a wide range of tumor cells, and it may have sensitizing actions in anti-cancer drugs [17,18]. The impact of lidocaine on miR-30c expression in cSCC cells has not yet been reported. Therefore, in this study, we explored the role of miR-30c in cSCC progression, and we examined whether lidocaine can act via miR-30c to influence proliferation and cisplatin resistance of cSCC tumors.

Here, we supposed that lidocaine could inhibit the cisplatin resistance of cSCC cells by inducing miR-30c/SIRT1 signaling pathway. To begin to identify its anti-cancer mechanisms of action, the present study examined the effects of lidocaine on the proliferation and resistance of cSCC cells, as well as the potential role of miR-30c/SIRT1 in mediating those effects.

## Materials and methods

### Cell culture

Human benign epidermal keratinocyte cells (HaCaT) and human cSCC cell lines (A431, HSC-5, SCC13, and Tca8113) were provided by the China Center for Type Culture Collection (Wuhan, China). All cells were cultured in Dulbecco’s modified Eagle medium (DMEM; Invitrogen; Waltham, MA, USA) supplemented with 10% fetal bovine serum (Gibco, Grand Island, NY, USA), 100 mg/mL streptomycin, and 100 U/mL penicillin (Invitrogen) in a 5% CO_2_ incubator at 37°C.

### Cell treatments

To construct the cisplatin-resistant model, A431 cells were first treated with 1 mM cisplatin for eight weeks. Then, the cisplatin dose was increased by 0.2 mM every two weeks until a final concentration of 3 mM was reached. The cisplatin-resistant (A431-R) cells were cultured in cisplatin-free DMEM for two weeks prior to experiments. A431 and A431-R cells were then treated with different concentrations of cisplatin (2, 4, 8, 16, 32, 64, and 128 μM) or lidocaine (0, 1,5, and 10 mM) for 48 h.

### Cell transfection

MiR-30c mimic, miR-30c inhibitor, and their respective negative control miRNAs (NC mimic or NC inhibitor), as well as short hairpin RNA against SIRT1 (shSIRT1, 5’-AACCTTCTGTTCGGTGATGAAA-3’, 487–505) and scrambled shRNA (shNC) were obtained from Invitrogen. The full length sequence of *SIRT1* was cloned into a pcDNA3.1 plasmid (Invitrogen) to produce a pcDNA3.1-SIRT1 construct overexpressing *SIRT1*. The empty pcDNA3.1 vector was used as negative control. A431 or A431-R cells were transfected with the prepared plasmids for 24 h using Lipofectamine 2000 (RiboBio Co., Ltd., Guangzhou, China).

### Cell viability

Cell viability was assessed using the cell counting kit-8 (CCK-8) assay (Invitrogen; Waltham, MA, USA). After transfection, A431 or A431-R cells were seeded into 96-well plates at a density of 5 × 10^4^ cells/well and incubated overnight at 37°C in a 5% CO_2_ atmosphere. The cells were then treated with different cisplatin or lidocaine concentrations for indicated times, and each well was supplemented with 10 μL CCK-8 solution, followed by incubation at 37°C for 2 h. Optical density (OD) at 450 nm was measured using a microplate reader (Bio-Rad, Hercules, CA, USA).

### Cisplatin resistance

The resistance of A431 or A431-R cells to cisplatin was determined by the CCK-8 assay. Briefly, A431 or A431-R cells were seeded into 96-well plates at a density of 5 × 10^4^ cells/well and incubated overnight at 37°C. After treatment with increasing cisplatin doses for 48 h, 10 μL CCK-8 solution was added to each well, followed by incubation at 37°C for 2 h. The respective IC_50_ values were determined using GraphPad Prism 5.0 software (GraphPad Software, La Jolla, CA, USA) and linear regression analysis.

### Cell counting

After transfection, A431 or A431-R cells were seeded into 96-well plates at a density of 5 × 10^4^ cells/well and incubated overnight at 37°C in a 5% CO_2_ atmosphere. The cells were then treated with different cisplatin or lidocaine concentrations for indicated times. After the indicated transfection or treatment, the number of viable cells was measured by a Coulter cell counter (Bio-Rad).

### Quantitative real-time polymerase chain reaction (qRT-PCR)

Total RNA was extracted from A431 cells using TRIzol reagent (RiboBio Co., Ltd.) and reverse-transcribed into cDNA using RNase-free RQ1 DNase (Takara Biotechnology Co., Ltd., Dalian, China). Levels of miR-30c and *SIRT1* mRNA were quantified using SYBR® Premix Ex Taq™ (Takara Bio Inc., Kusatsu, Japan) on an ABI PRISM 7500 Sequence Detection System (Applied Biosystems, Foster City, CA) following the manufacturer’s instructions. All samples were examined in triplicate using a Bio-Rad CFX-96 real-time cycler and the following primers: miR-30c forward, 5’-GCCGCTGTAAACATCCTACACT-3’; miR-30c reverse, 5′-GTGCAGGGTCCGAGGT-3′; SIRT1 forward, 5′-GACCTGAACTGCAGCAAGCCT-3′; SIRT1 reverse, 5′-CGTTCGAATAGCTAGTACGTCA-3′; β-actin forward, 5′-AGAACTGGCCCTTCTTGGAGG-5’; β-actin reverse, 5′-GTTTTTATGTTCCTCTATGGG-3′; U6 forward, 5′-CTCGCTTCGGCAGCACATATACT-3′; and U6 reverse, 5′-ACGCTTCACGAATTTGCGTGTC-3′. The relative levels of miR-30c and *SIRT1* mRNA were analyzed using the 2^−ΔΔCt^ method and normalized, respectively, to levels of β-actin mRNA and U6 RNA.

### Western blot analysis

Western blot was performed as describing in previous study [19]. After the indicated treatment or transfection, proteins were extracted using RIPA lysis buffer (Promega, Fitchburg, WI, USA) on ice for 30 min. After centrifugation at 12,000 *g* and 4°C, the supernatant was recovered and the total protein concentration was measured using a Coomassie protein assay kit (Promega). Equal amounts of protein were fractionated by 12% sodium dodecyl sulfate–polyacrylamide gel electrophoresis and transferred to nitrocellulose membranes. Membranes were then treated with 5% nonfat milk for 1 h to block nonspecific binding sites, and incubated with primary antibodies (Abcam, Cambridge, UK) against SIRT1 (ab30214) and β-actin (ab112014) at 4°C overnight, followed by incubation with secondary antibody (ab5027) at room temperature for 2 h. Antibody binding was determined by enhanced chemiluminescence (Millipore, Billerica, MA, USA).

### Dual-luciferase reporter assay

The potential binding sites of miR-30c in the 3’-UTR of the *SIRT1* gene were predicted using the starBase database and ligated to luciferase reporter vector pGL3 following the manufacturer’s instructions. A431 or A431-R cells were then co-transfected with lentiviral plasmids encoding pcDNA3.1-*SIRT1* containing the wild-type (WT) 3’-UTR or a 3’-UTR with mutations (Mut) eliminating the miR-30c binding sites, and with lentiviral plasmids encoding miR-30c mimic or NC mimic. Transfections were performed using Lipofectamine 2000. After culturing for 24 h, the cells were decomposed and the luciferase activity was measured using a dual luciferase reporter assay (Abcam, Cambridge, UK) following the manufacturer’s protocol. The relative luciferase activity was defined as the ratio of firefly to *Renilla* luciferase activity

### Statistical analysis

Statistical analysis was performed using GraphPad Prism 8.0 software. Data were expressed as mean ± standard deviation (SD). Each experiment was replicated three times. Differences among groups were evaluated for significance using Student’s *t*-test and one-way analysis of variance. Differences associated with *P* < 0.05 were considered statistically significant.

## Results

In our study, we supposed that lidocaine could suppress the cisplatin resistance of cSCC cells by inducing miR-30c/SIRT1 signaling pathway. To confirm the anti-cancer effects of lidocaine, we first observed its effects on the growth of cSCC cells. Then the potential roles of miR-30c/SIRT1 signal transduction in cSCC cells were observed.

### MiR-30c expression is significantly reduced in A431-R cells

**Figure 1.**
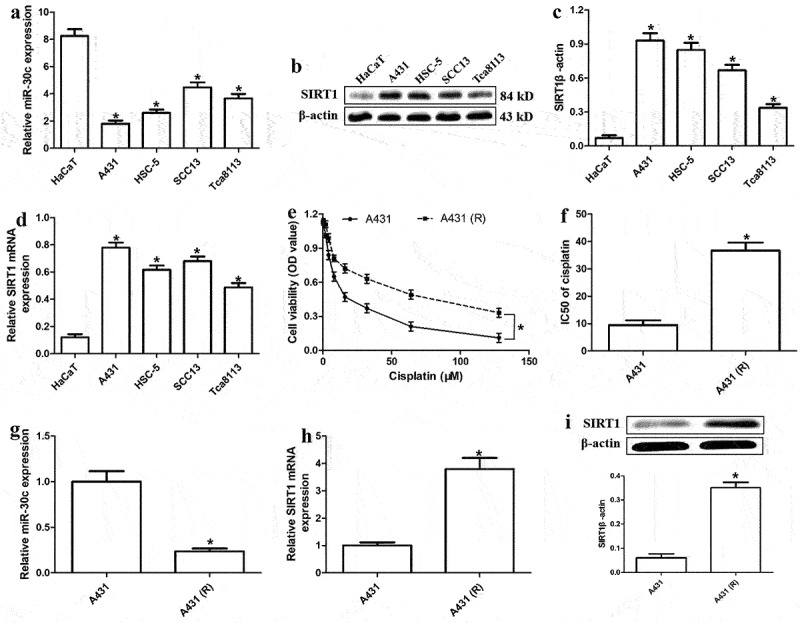
MiR-30c is significantly downregulated in cisplatin-resistant cutaneous squamous cell carcinoma (cSCC) cells. (a) Relative expression of miR-30c in HaCaT and cSCC cells. (b) Representative Western blot for SIRT1. (c) Relative protein levels of SIRT1 (normalized to levels of β-actin) in HaCaT and cSCC cells. (d) Relative *SIRT1* mRNA expression in HaCaT and cSCC cells, as determined by quantitative real-time polymerase chain reaction (qRT-PCR). (e) Viability of A431 and A431-R cells treated with different cisplatin concentrations (2, 4, 8, 16, 32, 64, and 128 μM) for 48 h. (f) IC_50_ values of cisplatin in A431 and A431-R cells, as determined from cell viability curves. (g-h) Relative levels of miR-30c and *SIRT1* mRNA in A431 and A431-R cells, as determined by qRT-PCR. (i) Relative expression of SIRT1 protein in A431 and A431-R cells. *P < 0.05, compared to HaCaT or A431 cells. A431-R, cisplatin-resistant A431 cells; HaCaT cells, human benign epidermal keratinocyte cells; OD, optical density; SIRT1, Sirtuin 1.

To explore whether miR-30c is associated with cSCC progression, we assessed its expression levels in HaCaT and the four cSCC cells A431, HSC-5, SCC13, and Tca8113. MiR-30c expression was significantly lower in cSCC cells than in HaCaT cells (Figure 1a), whereas the levels of SIRT1 protein and mRNA were significantly higher in cSCC cells than in normal cells (Figure 1b-d).

Since chemoresistance is closely related to tumor development in humans [16], we also explored the effect of miR-30c on chemoresistance in cisplatin-resistant cells. Compared to A431 cells, A431-R cells showed lower cisplatin sensitivity (Figure 1e) and significantly higher cisplatin IC_50_ value (figure 1f). In addition, miR-30c was significantly downregulated in A431-R cells compared to A431 cells, while SIRT1 expression showed the opposite trend (Figure 1g-i). Our results suggest that miR-30c underexpression is associated with cisplatin resistance and SIRT1 upregulation in cSCC.

### MiR-30c suppresses proliferation and cisplatin resistance of A431 cells

**Figure 2.**
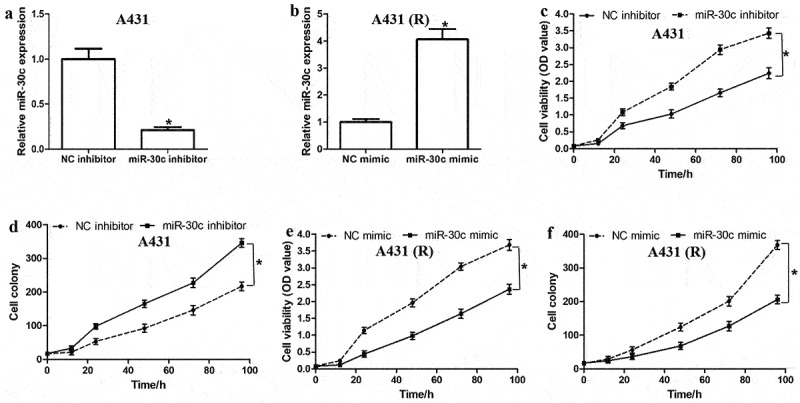
MiR-30c represses cell proliferation and cisplatin resistance in cutaneous squamous cells carcinoma cells. (a,b) Relative miR-30c expression in (a) A431 and (b) A431-R cells transfected with miR-30c inhibitor or miR-30c mimic, respectively. (c–f) Cell viability and colony numbers in (c,d) A431 and (e,f) A431-R cells transfected with miR-30c inhibitor or miR-30c mimic, respectively. *P < 0.05, compared to control group (NC inhibitor or NC mimic). A431-R, cisplatin-resistant A431 cells; OD, optical density.

To investigate the effect of miR-30c on cell growth and cisplatin resistance, A431 and A431-R cells were transfected, respectively, with miR-30c inhibitor or miR-30c mimic (Figure 2a-b). MiR-30c inhibition in A431 cells promoted cell proliferation (Figure 2c) and colony formation (Figure 2d). MiR-30c overexpression in A431-R cells had the opposite effects (Figure 2e and f). Our results suggest that miR-30c suppresses cell proliferation, colony formation, and chemoresistance in cSCC cells.

## *MiR-30c directly targets* SIRT1 *in A431 cells*

**Figure 3.**
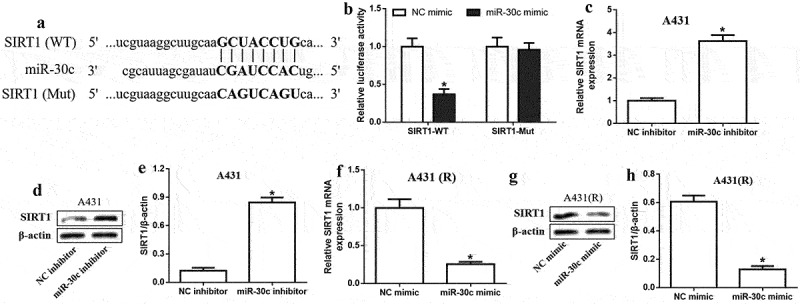
MiR-30c directly targets *SIRT1* in cutaneous squamous cell carcinoma cells. (a) Binding sites of miR-30c in the *SIRT1* 3’-UTR. (b) Relative luciferase activity in A431 cells expressing SIRT1-WT or SIRT1-Mut transcripts after transfection with NC mimic or miR-30c mimic. (c-e) Relative *SIRT1* mRNA and protein expression in A431 cells transfected with miR-30c inhibitor, as determined by quantitative real-time polymerase chain reaction and Western blotting. (f-h) Relative *SIRT1* mRNA and protein expression in A431(r) cells transfected with miR-30c inhibitor, as determined by quantitative real-time polymerase chain reaction and Western blotting. *P < 0.05, compared to control group (NC inhibitor or NC mimic). A431-R, cisplatin-resistant A431 cells; Mut, mutated; SIRT1, Sirtuin 1; WT, wild type.

To elucidate the potential mechanism of miR-30c, the interaction between miR-30c and *SIRT1* mRNA was explored. Potential binding sites of miR-30c in the *SIRT1* 3’-UTR were identified using the starBase database (Figure 3a). MiR-30c overexpression reduced luciferase activity in A431 cells expressing *SIRT1* mRNA with the WT 3’-UTR, but had no effect on cells expressing the SIRT1-Mut transcript (Figure 3b), suggesting that miR-30c directly targets SIRT1 in cSCC cells. Further experiments showed that miR-30c inhibition significantly upregulated SIRT1 at the mRNA and protein levels (Figure 3c-e), whereas transfection with miR-30c mimic had the opposite effects (figure 3f-h). Our results suggest that miR-30c can inhibit cSCC by directly targeting and downregulating SIRT1.

### SIRT1 antagonizes the effects of miR-30c on proliferation and cisplatin resistance of cSCC cells

**Figure 4.**
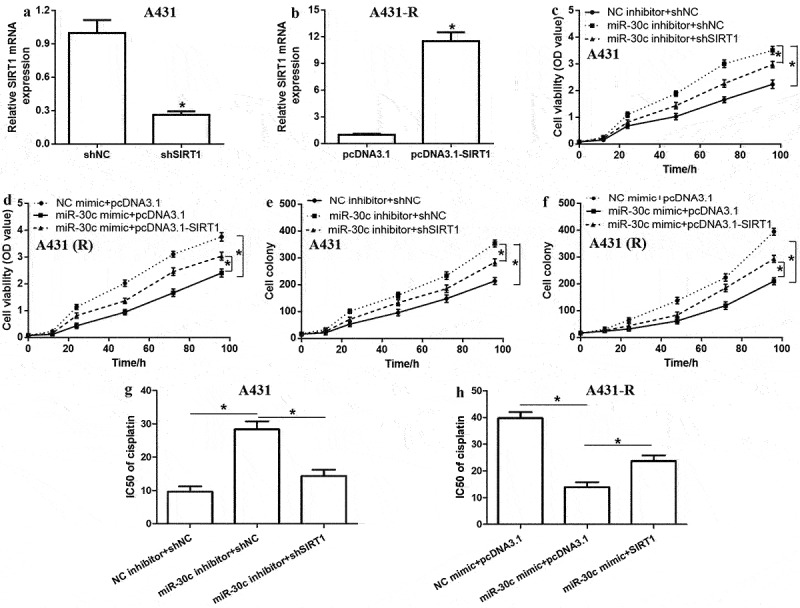
SIRT1 antagonizes the effects of miR-30c on cutaneous squamous cell carcinoma cell proliferation and cisplatin resistance. (a,b) Relative expression of *SIRT1* mRNA in (a) A431 and (b) A431-R cells, as determined by quantitative real-time polymerase chain reaction. (c–f) Cell viability and colony numbers in (c,e) A431 and (d,f) A431-R cells under- or over-expressing miR-30c after transfection with shSIRT1 or pcDNA3.1-SIRT1. (g-h) IC_50_ values of cisplatin in A431 and A431-R cells, as determined from cell viability curves. *P < 0.05. A431-R, cisplatin-resistant A431 cells; OD, optical density; shNC, scrambled shRNA; shSIRT1, short hairpin RNA against SIRT1; SIRT1, Sirtuin 1.

To further explore the role of the miR-30c/SIRT1 pathway in cSCC cell proliferation, we prepared A431 and A431-R cells that under- or over-expressed miR-30c and were transfected with shSIRT1 or pcDNA3.1-SIRT1, respectively (Figure 4a-b). Cell viability assays revealed that SIRT1 downregulation antagonized the ability of miR-30c underexpression to promote cell proliferation and colony formation in A431 cells (Figure 4c and e). In contrast, SIRT1 upregulation antagonized the suppressive effect of miR-30c overexpression on cell proliferation and colony formation in A431-R cells (Figure 4d and f). Furthermore, SIRT1 depletion weakened the ability of miR-30c underexpression to promote cisplatin resistance in A431 cells, whereas SIRT1 upregulation partly reversed the ability of upregulated miR-30c to reduce cisplatin resistance in A431-R cells (Figure 4g-h). These results confirm that miR-30c attenuates cSCC progression by inhibiting SIRT1 expression.

### Lidocaine represses proliferation and cisplatin resistance of cSCC cells by activating the miR-30c/SIRT1 pathway

**Figure 5.**
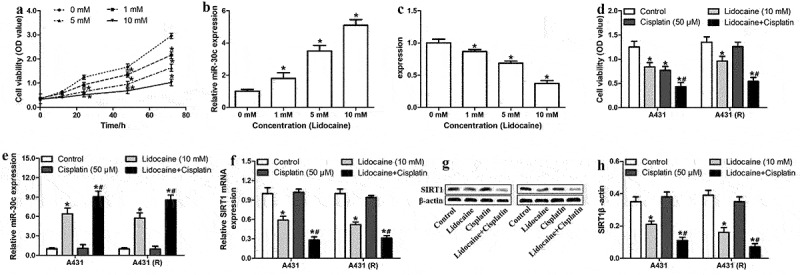
Combined treatment with lidocaine and cisplatin significantly reduces proliferation and chemoresistance in cutaneous squamous cell carcinoma cells. (a) Viability of A431 cells treated with increasing lidocaine concentrations for 12, 24, 48, and 72 h. (b,c) Relative expression of miR-30c and *SIRT1* mRNA in A431 cells treated with increasing lidocaine concentrations for 48 h, as determined by quantitative real-time polymerase chain reaction. (d) Cell viability, (e) relative miR-30c expression, (f) relative *SIRT1* mRNA expression and (g-h) SIRT1 protein in A431 and A431-R cells treated for 48 h with 10 mM lidocaine, 50 μM cisplatin, or their combination. *P < 0.05, compared to 0 mM lidocaine; ^#^*P* < 0.05, compared to 10 mM lidocaine or 50 μM cisplatin. A431-R, cisplatin-resistant A431 cells; OD, optical density; SIRT1, Sirtuin 1.

To clarify whether lidocaine attenuates cSCC progression and represses cisplatin resistance by regulating the miR-30c/SIRT1 pathway, A431 cells were treated with different lidocaine concentrations for different times. Our results indicated that lidocaine strongly suppressed A431 cell proliferation in a dose- and time-dependent manner (Figure 5a), while significantly upregulating miR-30c and downregulating SIRT1 in a dose-dependent manner (Figure 5b-c).

The synergistic effect of lidocaine and cisplatin on cell proliferation and the miR-30c/SIRT1 signaling pathway was also explored. Compared to the control group, treatment with cisplatin alone did not significantly affect cell proliferation or levels of miR-30c or SIRT1 in A431-R cells (Figure 5d-h). In contrast, the combined application of lidocaine and cisplatin significantly reduced proliferation of A431 and A431-R cells, while upregulating miR-30c and downregulating SIRT1. These results suggest that lidocaine represses cell proliferation and cisplatin resistance in cSCC cells by activating the miR-30c/SIRT1 pathway.

### MiR-30c underexpression antagonizes the ability of lidocaine to repress A431-R cell proliferation

**Figure 6.**
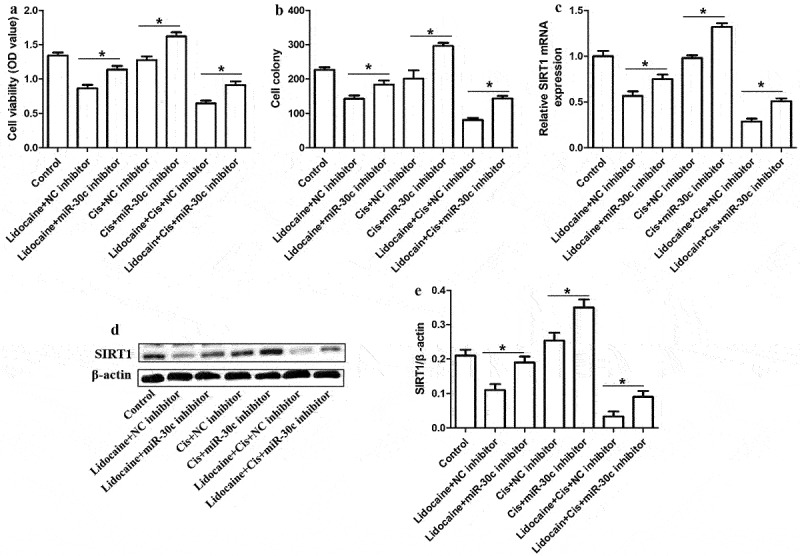
MiR-30c underexpression antagonizes the ability of lidocaine to suppress cell growth and chemoresistance in A431-R cells. (a) Cell viability and (b) colony numbers in A431-R cells transfected with NC inhibitor or miR-30c inhibitor for 24 h and subsequently treated for 48 h with 10 mM lidocaine, 50 μM cisplatin (Cis), or their combination. (c) Relative *SIRT1* mRNA expression, as determined by quantitative real-time polymerase chain reaction. (d-e) Relative SIRT1 protein expression, as determined by Western blotting. A431-R cells, cisplatin-resistant cells; OD, optical density. *P < 0.05.

To clarify whether lidocaine represses cSCC cell proliferation and chemoresistance by regulating miR-30c expression, A431-R cells were transfected with miR-30c inhibitor. Compared to the control group, treatment with lidocaine alone or in combination with cisplatin significantly reduced cell proliferation and colony formation and suppressed *SIRT1* mRNA expression. In contrast, treatment with cisplatin alone did not significantly affect cell growth or *SIRT1* mRNA expression. Downregulation of miR-30c partly reversed the effects of both lidocaine and cisplatin (Figure 6), suggesting that lidocaine inhibits cell proliferation and cisplatin resistance by upregulating miR-30c.

### SIRT1 overexpression antagonizes the ability of lidocaine to repress A431-R cell proliferation

**Figure 7.**
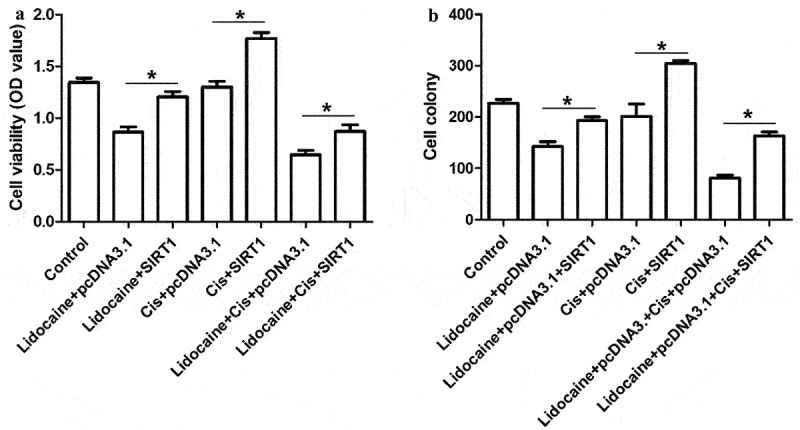
SIRT1 overexpression antagonizes the ability of lidocaine to suppress cell growth and chemoresistance in A431-R cells. (a) Cell viability and (b) colony numbers in A431-R cells transfected with pcDNA3.1-SIRT1 or empty vector for 24 h and subsequently treated for 48 h with 10 mM lidocaine, 50 μM cisplatin (Cis), or their combination. A431-R cells, cisplatin-resistant cells; OD, optical density. *P < 0.05.

To clarify whether lidocaine represses A431-R cell proliferation by regulating the expression of SIRT1, A431-R cells were transfected with pcDNA3.1-SIRT1 plasmid. Treatment with lidocaine alone or in combination with cisplatin significantly reduced cell proliferation and cell colony formation in A431-R cells compared to the control group (Figure 7). In contrast, cisplatin alone did not significantly affect cell growth. SIRT1 overexpression partly reversed the effects of lidocaine and cisplatin, indicating that lidocaine inhibits cell proliferation and cisplatin resistance in A431-R cells by downregulating SIRT1.

## Discussion

The second most frequent cancer worldwide [19], cSCC is a keratinocyte-derived malignant skin tumor that is associated with high recurrence and metastasis rates, leading to increased mortality [20]. Although cisplatin is considered a potent platinum-based chemotherapeutic drug for cSCC treatment, its clinical application is limited due to the high chemoresistance of cancer cells [21,22]. In the present study, we found that miR-30c can suppress cell proliferation and cisplatin resistance in A431 cells and protect against cSCC progression by directly targeting and inhibiting SIRT1 expression. In addition, we found that lidocaine can effectively suppress A431 cell proliferation in a dose- and time-dependent manner. Further experiments on the effect of miR-30c underexpression and SIRT1 overexpression in A431-R cells confirmed that lidocaine can suppress cell proliferation and cisplatin resistance in cSCC cells *via* the miR-30c/SIRT1 pathway. Our results not only demonstrate the anticancer properties of lidocaine, but they also highlight miR-30c as a promising therapeutic target in cSCC.

MiRNAs are involved in the regulation of drug resistance in many types of cancer. For example, miR-218 attenuates the resistance of gastric cancer cells to cisplatin by targeting survivin [23], while miR-142-5p promotes cisplatin-induced apoptosis in ovarian cancer cells by targeting multiple anti-apoptotic genes [24]. Moreover, miR-34a-5p has been found to modulate cisplatin resistance in ovarian cancer cells by downregulating programmed cell death 1 ligand 1 [25]. MiR-30c has been associated with antitumor activity in many human malignancies. For instance, endothelial miR-30c repressed survival of tumors by inhibiting TGF-beta-induced Serpine1 [9], while it attenuated growth of colorectal carcinoma tumors by directly targeting BCL9 [26], and it reduced proliferation and migration of glioblastoma cells by directly targeting SOX9 [27]. The biological functions of miR-30c in the development of cSCC have not yet been elucidated. Here, we found that miR-30c is underexpressed in cSCC cells and even more so in cisplatin-resistant cSCC cells, suggesting that miR-30c is closely associated with cisplatin resistance in cSCC. Further experiments confirmed that miR-30c overexpression repressed cell proliferation and cisplatin resistance in cSCC cells by downregulating SIRT1.

SIRT1 is involved in aging, energy metabolism, and autophagy [28]. The enzyme deacetylates proteins and modulates their transcription or activity, thus regulating cellular energy metabolism and stress response signaling pathways [29]. SIRT1 overexpression regulates doxorubicin resistance in breast tumor by activating the Akt pathway [30], and SIRT1 promotes tumor growth in colorectal [31], breast [32], and pancreatic cancers [33]. Consistent with these studies, we found here that SIRT1 is significantly upregulated in cisplatin-resistant cSCC cells and antagonizes the effects of miR-30c on cell proliferation and chemoresistance.

Lidocaine, a local anesthetics, and is closely associated with skin injury and can modulate the expression of various miRNAs [^34–36^]. Lidocaine has been reported to inhibits hepatocellular carcinoma development via regulating miR-421/CPEB3 pathway [37], and inhibits glioma cell proliferation, migration and invasion via regulating the circEZH2/miR-181b-5p axis [38]. Furthermore, lidocaine alleviates cisplatin resistance and inhibits migration of lung cancer and gastric cells through the decreasing miR-21 and miR-10b, respectively [39,40]. In this study, we found that lidocaine can suppress cell proliferation and cisplatin resistance in cSCC cells by upregulating miR-30c and downregulating SIRT1. We confirmed these results by showing that miR-30c underexpression and SIRT1 upregulation counteracted the effects of lidocaine.

## Conclusion

This study supports that miR-30c can suppress cSCC progression by directly targeting and repressing the expression of SIRT1. Our results not only provide new insights into the role of miR-30c in cSCC pathogenesis, but they may also be useful for developing effective treatments against cSCC. Indeed, our findings support the therapeutic potential of lidocaine against proliferation and cisplatin resistance in cSCC. In the future, we will apply the nanoparticles for delivery of lidocaine to study its anti-tumor activity.

## Highlights

- MiR-30c targeting and downregulating SIRT1.
- MiR-30c overexpression also suppressed cisplatin resistance in A431 cells.
- Lidocaine suppressed A431 cell proliferation and cisplatin resistance.
- Lidocaine activates the miR-30c/SIRT1 pathway.
